# Progress of Veterinary Science

**Published:** 1883-10

**Authors:** 


### Progress of Veterinary Science.

A New Parasiticide.—Guerin, (Th. de Paris 1882,) recommends napthol as a parasiticide in *l'acarisse auriculaire* among dogs.

Cattle Liable to Abort.—Dr. John Faust, V.S., in the *American Veterinary Review*, recommends the use of *viburnum prunifolium* for abortion in cattle. Dose—One-half drachm every day in cases of infection. Threatened abortion, same dose every hour or two as the case may require, and confine in large box stall.

Itch in the Cat.—A correspondent of the *British Medical Journal*, Dr. John Reid, writes as follows regarding a case of "acarus" in a cat: "The cat in question, when seen for the first time (it being a stray cat), was greatly emaciated, and died on the following night (January 5th, 1883). The hair on one side of the face and neck, including the ear, was matted so as to resemble one large scab. The itch-insect and eggs were detected in large numbers. The cat's liver contained many abscesses of the size of a pin's head; the lungs, etc., appeared to be normal. Does the cat infect children, etc.? do these infect the cat? or is there mutual infection?"—*Medidal Record.*

Amputation of Penis.—The patient was a waler in a horse artillery battery, aged nine years, which had a very large barsatti growth on the end of the penis. It was about being destroyed, but as it was the best looking horse in the battery I determined to operate. Having cast and secured my patient, I detailed the salootrie to administer chloroform. I could not pass the catheter, for the growth surrounded the opening of the urethra completely, so I bound a piece of strong tape close to where I was going to make my first incision, and having entrusted this to the hand of a shoeing smith, I began work. I tried torsion on the vessels, but failed to stop hœmorrhage, so I ligatured every one with silk. He did service afterwards in Afghanistan. —*Quarterly Journal of Veterinary Science in India.*

Chicken Cholera.—A chicken died on a farm at Gers last year of cholera, which had been epidemic there. It had laid several eggs before death which I submitted to incubation, marking them so as to distinguish them from others of the hatch. No difference could be detected between the marked and unmarked eggs until the circulation of the allantoid commenced between the eighth and the tenth day of their embryonic development. At this stage the marked eggs ceased to progress. On opening we found on the surface of the allantoid a lake of black blood, having an odor peculiar to the diseased fowl. The umbilical artery continued to pulsate for some time, showing that life is slow to become extinct in the embryo. The latter was found at the bottom of the amniotic pocket gorged with a large quantity of liquid, whilst all the albumen had disappeared. The blood was filled with bacteria and the amniotic fluid contained extremely small monads. It is evident that the eggs contained the germs of microbes with which the blood of the mother had been gorged and that these germs are not developed, but at the period of ærial respiration when the allantoid has transmitted to the blood the necessary oxygen for the development of the bacteria. It is interesting to remark that it is only at this moment that the embryo really presents the character of the bird.

I made three chickens swallow the remains of these embryos and two have already succumbed. It is true that the disease still reigns on the farm and that other chickens are attacked.—*M. A. Barthélemy in Annals et Bulletin de la Société de Médecine de Gand.*

Fracture of Femur in a Puma.—Mr. Henry Morris showed this specimen for Mr. Sutton of the Zoölogical Society's Gardens. The right thigh had been the seat of an old injury; the acetabulum was occupied by a mass of fibrous tissue; the femur was displaced, and the head of the bone was probably represented by a small bony mass attached to the ilium by ligaments, and still freely movable.

Pneumothorax in a Coati.—This speciman came from the same source. The pleura presented the ordinary appearances of pneumothorax as seen in man. Another coati had recently died in the gardens from the same condition.

Acute Farcy in Man.—Mr. Howard Bendall (Birmingham) showed some microscopical sections, and read the notes of the case, which were briefly as follows: The patient, an adult male, was admitted into hospital suffering from an erysipelatous condition of the right foot, and from a number of swellings, varying in size from a pigeon's to a turkey's egg, scattered over the limbs. Two of these swellings, softer than others, were opened; and the pus evacuated was found to contain a considerable quantity of free oil. Three days later the characteristic pustular skin-eruption appeared, and there was nocturnal delirium. A couple of days later, some pus was seen in the sputum and there were physical signs of pneumonia at the left base. The dyspnœa, which was now evident, rapidly increased, and seemed to be out of all proportion to the physical signs. Two days later, a thin yellowish acrid fluid commenced to exude from the nostrils. Eight days after admission he died comatose. The necropsy was made twenty-four hours after death. The blood was dark and fluid; but the right ventricle contained a large partially decolorized clot, which extended along the pulmonary arteries. The lower lobe of the left lung contained three abscesses, one of which had burst into a bronchus; the base of this lung was consolidated. Both lungs showed bronchitic changes. There was no ulceration of larynx, trachea, or bronchi. In the nasal cavity and on the roof of the mouth were numerous ulcers; these were covered, some with sloughs, others with a foul mucopurulent discharge. Some of the ulcers extended on to the fauces and pharynx. The spleen was enlarged and pulpy; but the other abdominal viscera were healthy. The left tunica vaginalis was obliterated by adhesive inflammation, and a few purulent points were seen on section of the testicle. Scattered throughout the muscles of the limbs, and more especially in the neighborhood of the joints, were large numbers of abscesses, containing sloughy blood-stained pus; in all, there were forty or fifty such abscesses. The skin of the chest and forehead was scattered over with small pustules; some of these had burst, leaving ulcers from one-eighth to one-fourth of an inch in diameter. Examination of microscopical sections of the lung from the neighborhood of the pneumonic nodules showed that the arterioles were more or less completely plugged with oil, which also enormously distended some of the capillary vessels. The mucous membrane of the hard palate had undergone intense disintegrative inflammation. In some regions, chiefly near the side or floor of the ulcers, granules of fat could be seen in clusters. The skin of the forehead in the region of the ulcers was in a similar condition. The sebaceous glands seemed to have undergone extensive fatty necrosis. Mr. Bendall pointed out that it was of great interest to notice the concurrence of this free oil in the abscesses, and of this curious form of fatty degeneration of the skin and mucous membrane, with the fat embola in the lungs. Dyspnœa appeared to be an almost constant symptom in the latter stages of acute farcy, and might own this origin.—*British Medical Journal.*

Sarcoma in a Common Fowl.—Malignant growth is probably common enough in birds, but as indubitable cases are not often described, the following account of the development of a sarcomatous tumor in the neck of a common hen may prove of interest.

The bird was hatched in the spring of 1880, and a year ago a swelling was first noticed growing from the neck immediately below the beak. This growth, when I first saw it in January, 1883, was a little larger than a cricket ball; it was perfectly distinct from the crop, and freely moveable; it was very heavy, so that its weight prevented the bird from raising the head or flying from the ground; it weighed six ounces and three-quarters when removed. The skin adhered on the under surface, on account of the irritation and hardening produced by contact with the earth. I removed it under chloroform on January 26th, and brought the wound together, which healed by first intention. During the week following, the bird gained seven ounces in weight, and soon recommenced laying eggs; this had stopped entirely during the year's growth of the tumor. About the middle of May she hatched a brood of healthy chickens produced from her own eggs. Two months after the removal of the growth the disease recurred in the scar, and the glands round became rapidly affected. Mr. Bowlby, of St. Bartholomew's Hospital, has been kind enough to make a microscopic examination of the part removed, and he says that "it consists of round cells, each about the size of a human red blood-corpuscle, without any definite cell-body, and somewhat granular. There was no appearance indicating an alveolar arrangement; but scattered irregularly amongst the cells was a little fibrillated tissue. There can be no doubt of the malignant nature of the growth, which seems to belong to the sarcomata rather than the true cancers or carcinomata."—*British Medical Journal.*

The Principal Diseases of the Hock.—At the May meeting of the Midland Counties Veterinary Association, Professor Walley delivered a lecture on the above subject. He remarked at the outset that the subject was of very great importance to those who examined horses for soundness, led to much controversy among Veterinarians, and therefore, thought it a proper one to be discussed. He went on to say that of all the diseases of the hock the so-called bone spavin was the most important, and he showed that the formation of bony deposits on the internal aspect of the joint was caused by the peculiar anatomical arrangement of the bones. But while he desired to point out particularly with reference to the formation of these bones the existence of a very marked predisposing cause for spavin, he mentioned hereditary predisposition as another very strong cause, remarking that of all animals with which they had to deal, the horse was the most predisposed to bony deposits and especially of the hock. He considered that one of the greatest predisposing causes of spavin was the peculiar manner in which the weight was thrown upon the small cuneiform bones of the inside of the hock, the articulation here differing very materially from that on the outside of the joint. The pathology of the disease was inflammation with exhudation of bony matter. The chief symptom was lameness marked by a few peculiarities. The lameness disappeared with exercise; the horse was most lame on turning on the sound leg; the toe or shoe was worn, more frequently on the outside than on the inside, caused by the animal endeavoring to throw the weight on the inside of the foot. Often no symptom presented itself and then a careful examination of the hock was necessary for the detection of spavin. He pointed out the rules observed in manipulating the joint for the purpose, and thought taking a mould of the hocks perfectly justifiable. There was a difference between spavin and what was known as coarse hocks, and his rule in dealing with them was to contrast them one with the other and take all the bones and the age of the horse into consideration. If they found all the bones in keeping, and those perfectly free from lameness he thought they were justified in passing the animal sound; but, on the other hand, if they found one joint bigger than the other, even if the horse was apparently perfectly sound, they were not justified in passing him without a remark. Treatment—Counter-irritation; in firing always force the point of the iron into the bony structure.

The Professor next described the characteristics of occult spavin. Although the cause of this disease was somewhat doubtful, he attributed its origin to the peculiar motion of the joint and the consequent material interference with the blood supplying that region. In this form of the disease the lameness did not pass off with exercise. There was no enlargement of the joint. The temperature was high and concussion caused pain. The treatment consisted in producing anchylosis which was not always easy to do. In gouty disease of the hock, to which heavy cart horses employed in town work were liable, he recommended a change of food and the external application of iodine. He next spoke of spavin and throughpin and the necessity of diagnosing between them. Curb he defined to be a thickening of the ligament sometimes caused by striking the joint. He mentioned a case in which a horse was refused the prize for the best stock getter because he had been fired and the official veterinary surgeon very properly refused to pass him, although the injury was due to an accident. The result proved that the veterinary surgeon was right, for afterwards twenty colts by the horse might have been found all with curby hocks.—*The Veterinarian.*

Treatment of Poll Evil.—Before pus is formed, and swelling is only apparent, I have found great benefit from applying a good strong iodine blister to the affected parts, together with the administration of a purgative. We are advised by eminent practitioners to first use cold water applications, *i. e.*, in the primary stage. I must admit I never think of cold water treatment in cases of this kind. My first thought is just what I have just stated—applying a good blister over the part and if necessary repeat it.

If we are sure pus is formed, we cannot open the abscess too soon by making a good free cut with the knife, and don't be afraid of using it. The cut must be made longitudinally, following up the sinus to the very end if possible, taking care not to cut a muscle transversely, as, by so doing, we get what too often follows these cases—viz.: poking of the nose.

After laying the sinus well open, we generally dress the wound with antiseptic or digestive liniment, at the same time using every means in our power not to allow the opening to heal at the top first. This is in a great measure obviated by packing the wound with fine tow, first steeped in the above mentioned liniment, and I am sure I need not tell you how difficult it is to keep the tow in the wound. I may state that in packing the wound we should be very careful especially if it is a very deep one. I have heard of a late Veterinary Surgeon, who was well known to nearly all present, having a case of this kind, and in packing the wound the horse happened to throw up his head, and in an instant he (the horse) was *non est.* In fact our veterinary had pithed him. I simply mention this to show how careful we ought to be in such cases. We often find it necessary to keep down the most external granulations by the application of caustic.

In conclusion, I must say I do not believe in injecting caustics into the sinuses without first free use of the knife. If caustics are injected we find structure destroyed we ought by all means to try and preserve, especially the "ligamentum nuchæ."

I forgot to mention earlier on that in one case we resorted to the old fashioned practice of setons, and I must say it answered remarkably well.

Since I wrote this paper, three months ago, I have had two more cases, which were treated as I have stated above, *i. e.*, by opening the wounds longitudinally and dressing with digestives. One of the two cases has quite recovered. The other we have under treatment at the present time, and is doing well.—*Mr. Kitchen in the Veterinary Journal.*

Treatment of Quitters.—There are two things necessary in order to ensure a satisfactory result in Quitters; the first is bottom your sinus or sinuses; don't be frightened. Secure your horse firmly in the stocks, then go to work, and, to use a vulgar phrase, make a clean sweep. I don't believe in the continual use of arsenic, bichloride of mercury, or any such sloughing agents; trace the sinuses with your probe, find out how they go and how many there are; then with the hot iron take the whole piece out *holus bolus;* after which treat as a common wound, and if a sinus should form again, take it out in the same way. No doubt you will think this very heroic treatment, but I ask those of you who have not tried it, to do so, and I think you will find it to answer in all cases.

I happened to be in Yorkshire a few months ago, when, I met wi.a a very old friend of mine, a Veterinary Surgeon, and in the course of our conversation, Quitters were mentioned.

My friend told me he had been treating a case with iodoform and with very good results. In fact the case got well. He simply wet the probe, then getting it charged with the pulv. iodoform applied it to the sinuses, and he told me it was surprising the effect it seemed to have on the parts. I simply mention this in passing ; I have not tried it myself.

Now the second thing in the treatment of Quitters is this: don't be always probing and interfering with them. When you have thoroughly bottomed the sinuses, as before stated, and you are sure you have bottomed them, then leave the parts alone to nature.

We, as Veterinary Surgeons, interfere too much with our cases; we can't cure anything. We can only assist nature; but instead of assisting nature we retard her by our continual interference. I remember when a boy at home (which was not fifty years ago), my father had a mare with Quitters. The mare in question was attended to by a M.R.C.V.S. for some time, but without any apparent benefit. At last some old fellow turned up (I am sorry to say there are plenty of them, especially in country places), who undertook the case on the no-cure, no-pay system, and I assure you, in six weeks the mare was well. What he applied to the foot I don't know; it was a *liquia acca* or caustic of some sort. It is not what he applied to the wound that I wish to allude to, but the absolute rest he gave the parts afterwards, only coming to look at the mare once a week.

There is another thing I wish to mention before I close, and that is I don't believe in bandaging up Quitter wounds. If there is any pus forming, we prevent its escape, and, what is more, we keep from the wound one of the nicest and mildest astringents we have, viz.: atmosperic air.

I believe fifty per cent. of our Quitter cases if simply turned out to grass when it is seasonable, would recover of themselves.—*Mr. Kitchen in the Veterinary Journal.*

Cancer of the Stomach.—The subject of this case was a grey troop horse, 12 years of age, which had for the past year frequently suffered from mild attacks of colic. These generally lasted from three to twelve hours, On the 21st of May, 1883, he was brought to the sick lines, showing symptoms of abdominal pain. An anti-spasmodic draught was administered and appeared to give relief for a time. During the following night the patient experienced an attack of Acute Congestion of the Lungs, for which he was treated with stimulants internally, and applications of mustard to the throat, course of trachea and chest. Towards morning of the 22d, he seemed relieved, but during the day suffered from high fever, the breathing became abdominal and labored and there was a frequent distressful cough. Pleuritic complications were now detectible, and the treatment modified accordingly, consisting principally of fomentations and the daily administration of salts of potash. On the 28th, with a view of improving the appetite, tonics were given in gruel and during the following three weeks the patient seemed to improve slowly. On the 20th of June I was enable to detect consolidation of the posterior end of each lung. The treatment being continued he gradually improved and was discharged relieved, on the 5th of July. On the 8th, being reported dull and off feed, he was re-admitted, and the case entered as one of Debility. On this date I again carefully examined him by means of a stethescope and diagnosed emphysema of the superior part of both lungs; no sound whatever could be detected at the lower part of these organs; pulse regular but feeble; a most hard hacking cough was present, and the appetite very bad. Treatment: Tonics (vegetable and mineral alternated), general nursing and grazing daily. Remained much in the same state until the 9th of August, when he was recommended for casting. August 20th; had a slight attack of colic; an anti-spasmodic anodyne draught was adminstered which had the desired effect. September 1st; no improvement, lying down, apparently free from pain, pulse 40 and very weak. The animal died at an early hour next morning.

*Post Mortem Examination*—Intestines pallid, some gravel in the colon but no disease of its walls; liver, kidneys and spleen small. The posterior part of right lung consolidated and the upper margin emphysematous; the left lung fairly healthy. Both pericardial and plural sacs contained an abnormal amount of serous fluid; heart small and flabby. The coats of the stomach considerably thickened and diseased; total weight of the organ about seven pounds, specimen forwarded to V. S. J. H. Steel, for examination, who reports as follows:

"A very considerable portion of the lining membrane had undergone cancerous change, the villous portion being that mainly invaded. Throughout almost the whole of its extent it was thickened and broken up into cancerous masses or strings; the whole surface covered with soft fluid, carcinomatous material, distinguishable as such by its grumous character, and its component cells being evidently, *to the naked eye*, somewhat fusiform and inclined to adhere together in strings, formed by their overlapping one another at their ends. A thicken cancer discharge might be obtained by expression from cavities in the mucous membrane, which required to be cut open. One part of the villous membrane was so elevated as to form a semi-ovoid tumor, six inches by four extending in the long diameter of the organ. This elevated part did not seem to be invaded by the disease, but section showed that the tumor resulted from gelatinous deposit in the submucous tissue, which deposit, examined microscopically, was found to consist of round polynucleated carcinoma cells. In addition to the conditions hitherto mentioned, might be noted a strong band of cancer impregnated remnant of mucous membrane, forming a bridge connecting one side of the stomach with the other; also, here and there, small orange colored prominences, their color depending on small caps of rather degenerated epithelium covering them.—D. C. PALLIN, V· S., 14th Hussars.

REMARKS (by EDS.).—Apart from the advisabilitv of placing on record every case of indubitable cancer in the horse, we desire to draw attention to the complete absence of symptoms sufficient to ensure precise diagnosis of the seat and nature of disorder. This is very frequently the case in diseases of the stomach. We may give the following case in illustration of the same point:

THICKENING OF THE VILLOUS MEMBRANE OF THE STOMACH.—B. M., 4 years old; admitted February 1873, suffering from progressive Asthenia. Since she joined the regiment six months before, it had been noticed that she did not thrive. She passed numerous nematode worms, and her bowels were irregular. No impairment of the appetite in spite of all efforts of the V. S. of the regiment, the debility so increased that on the 17th of April she lost all sensory power of the hind limbs, but only partially the motor power. Her breathing became labored and pulse imperceptible; she died early on April 18th. Autopsy showed lungs congested; liver congested but small; heart large and flabby; stomach with practically the whole of its villous portion of the mucous membrane in a state of hyperthropyhy, with complete obliteration of its gastric follicles, but enormous increase in its fibrous and epithelial elements; muscular coat of the viscus unusually vascular, and sending very large blood vessels into the neighboring mucous layer.—*Quarterly Journal of Vet. Science, India.*

